# The genetic diversity of Ethiopian barley genotypes in relation to their geographical origin

**DOI:** 10.1371/journal.pone.0260422

**Published:** 2022-05-27

**Authors:** Surafel Shibru Teklemariam, Kefyalew Negisho Bayissa, Andrea Matros, Klaus Pillen, Frank Ordon, Gwendolin Wehner

**Affiliations:** 1 Ethiopian Institute of Agricultural Research (EIAR), Melkassa Agricultural Research Center, Melkassa, Ethiopia; 2 Ethiopian Institute of Agricultural Research (EIAR), National Agricultural Biotechnology Research Center, Holetta, Ethiopia; 3 Julius Kühn Institute (JKI), Federal Research Centre on Cultivated Plants, Institute for Resistance Research and Stress Tolerance, Quedlinburg, Germany; 4 Institute of Agricultural and Nutritional Sciences, Martin Luther University, Halle (Saale), Germany; Università Politecnica delle Marche, ITALY

## Abstract

Ethiopia is recognized as a center of diversity for barley, and its landraces are known for the distinct genetic features compared to other barley collections. The genetic diversity of Ethiopian barley likely results from the highly diverse topography, altitude, climate conditions, soil types, and farming systems. To get detailed information on the genetic diversity a panel of 260 accessions, comprising 239 landraces and 21 barley breeding lines, obtained from the Ethiopian biodiversity institute (EBI) and the national barley improvement program, respectively were studied for their genetic diversity using the 50k iSelect single nucleotide polymorphism (SNP) array. A total of 983 highly informative SNP markers were used for structure and diversity analysis. Three genetically distinct clusters were obtained from the structure analysis comprising 80, 71, and 109 accessions, respectively. Analysis of molecular variance (AMOVA) revealed the presence of higher genetic variation (89%) within the clusters than between the clusters (11%), with moderate genetic differentiation (PhiPT = 0.11) and five accessions were detected as first-generation migrants using Monte Carlo resampling methods. The Mantel test revealed that the genetic distance between accessions is poorly associated with their geographical distance. Despite the observed weak correlation between geographic distance and genetic differentiation, for some regions like Gonder, Jimma, Gamo-Gofa, Shewa, and Welo, more than 50% of the landraces derived from these regions are assigned to one of the three clusters.

## Introduction

Barley (*Hordeum vulgare* L.) ranks fifth in the acreage and production of cereals after tef, maize, wheat, and sorghum in Ethiopia. It accounts for 5.63% of the total cereal production (811,782.08 hectares (ha)) with a productivity of 2.18 ton/ha in 2018/19 [1]. It is a widely adapted crop, cultivated from drought prone lowlands of 1,500 meters above sea level to highlands of Ethiopia with an altitude of 3,400 meters above sea level with adequate moisture [2]. Most of the barley acreage is located in the altituted range of 2,400 to 3,400 meter above sea level in the northern and central part of the country [3]. In Ethiopia, barley is an important cereal crop grown by smallholder farmers for subsistence with limited capacity for modern agricultural practices, and in areas where soil fertility, drainage conditions, and topography are not suitable to produce other crops [4]. It is cultivated in two seasons; *‘meher’*, which is the major rainy season (June to October) in which diverse genotypes are grown, and *‘belg*’ with less amount of rain (late February to early July) in which most early maturing varieties are grown [5]. The total amount of barley production in *‘meher*’ is by far exceeding the one in *‘belg’*, which covered 84.5% of the total area of production and 93.0% of the total yearly barley harvest in 2013/14 [6].

The origin and domestication of barley believed to be ‘Fertile Crescent’ [7]. Ethiopia is recognized as a center of diversity for barley, as it is cultivated in a wide range of agro-ecology zones for centuries, and its landraces have exhibited distinct genetic diversity from the rest of the world’s barley collections [8–10]. Study using chloroplast SSR markers between Ethiopia/ Eritrean landraces and wild barley (*H*. *vulgare ssp*. *spontaneum*) revealed that the wild barley might not be an ancestor for barleys of Ethiopia/ Eritrean and these landraces might have different domestication path [11]. The presence of diversified and distinct genetic features have been explained by geographical isolation of the country from other barley growing regions for long periods together with the occurrence of diverse soil types, climate conditions, elevation, and landscape, which affect the type of farming system practices [12, 13]. One study indicated that Ethiopian barley population structure depends on the farming system, elevation, and barley row types [14]. Additionally, social factors like a preference of genotypes suited for different use also contributed significantly to the diversification [15]. Therefore, it was suggested that the diversity in Ethiopian barley landraces came due to a combination of long period accumulation of distant mutations, gene recombination, hybridization, natural selection, and human preference in a highly diversified agro-ecological environment [16].

The genetic resources of Ethiopian landraces are still rich and well maintained, as a report indicated that 95% of the Ethiopian smallholder farmers use landraces as the major seed source [17, 18]. Although barley is an inbreeding species with less than 5% of outcrossing, an increased rate of outcrossing was reported in Ethiopia, which is probably related to abiotic stress or variable environmental conditions [19]. Barley landraces at hand of farmers are genetically highly variable [20, 21], as farmers mainly focus to maintain morphologically uniform seeds than genetically uniform seeds, thus, sampling from smaller plots of farmers’ land may result in a collection of highly genetically diversified seeds [3].

Traditionally, farmers classified barley landraces based on kernel type as hulled, hull-less, and partially hulled barley [3]. Additionally, participatory research on durum wheat landraces revealed that farmers also considered yield, quality related to end-use products, and tolerance to different abiotic and biotic stresses like drought and diseases for the classification and selection of landraces [22]. Ethiopian barley landraces are particularly diverse in morphological appearance [23, 24] and bio-chemical composition, e.g. different hordein polypeptide patterns [25, 26] as well as anthocyanin coloration on seed coats, leaf sheath and stems [27].

The genetic structure of a population is influenced by variation in geographical collection distance, presence of geographical barriers like wetlands, mountains and gorges, as well as by the compatibility of genotypes to cross to each other. Besides this, the genetic structure is also due to the presence of barriers on the human local population over a long period of time [28].

Application of molecular tools improved the efficiency and precision of analysis of genetic relatedness in different crop species, as they helped to decipher whether the morphological, chemical, traditional and geographical classifications are in consistence to molecular structural analysis [29]. Different kinds of markers, i.e. AFLPs (amplified fragment length polymorphism), SSRs (simple sequence repeat), and SNPs (single nucleotide polymorphism) were used for genetic analysis of different cultivars, breeding lines and related species of barley [30–35]. Currently, SNP markers are commonly used to study genetic variation, as they are more abundant, with minimum mutation rate and have high-throughput performance than other markers, which enable to trace the important genome and its regions; ultimately empower the association of the markers with the interest of trait [36, 37]. The development of a 50k iSelect SNP array by [38] further enhanced the genetic exploration with accurate physical positions of the markers and detailed gene annotation.

The presence of genetic divergence between populations can be studied using Nei’s genetic distance [39]. Genetic abundance or richness within a population can be explored using the Shannon index [40, 41], whereas the variability within a population can be studied using heterozygosity indices [42]. The fixation index (F_ST_) is widely used to investigate the genetic distance between populations [43, 44]. As described by [45] indirect estimation of net migration rate using F_ST_, were likely to be true. Therefore, Monte Carlo resampling methods [46] is better to study first-generation migrants, while the gene flow between genotypes from different geographic locations can be studied using the Mantel test [47]. The neighbor-joining tree method is used to graphically demonstrate the distance between different genotypes based on their genetic background [48].

Several studies on the genetic distance of Ethiopian landraces using different molecular markers were conducted. Distinctive genetic features of Ethiopian landraces compared to other barley collections were reported, although a minimum genetic distance between different Ethiopian landraces was detected using RFPLs (restriction fragment length polymorphism) markers [49]. Another study revealed the presence of different levels of the allelic richness and genetic diversity in relation to altitude using seven SSR markers [21]. [50] also revealed a poor population structure for landraces collected from different regions of the country using 15 SSR markers. Genetic diversity studies of Ethiopian barley genotypes in relation to different world barley collections were also conducted using SSR [11], and AFLP markers [7] and the findings suggested Ethiopia as a second center of barley domestication.

Therefore, the aims of this study were, (i) to investigate the genetic diversity of Ethiopian barley landraces, and (ii) to analyses the role of the geographic origin, and defined agro-ecological zones in the formation of genetic structure using a highly informative 50k iSelect SNP array [38]. The outputs of the study will support the strategic collection and exploitation of existing barley genetic resources, to improve the livelihood of the subsistence farmers through strategic utilization of genetic resources available on the hand of smallholder farmers.

## Materials and methods

### Plant materials

**Fig 1:**
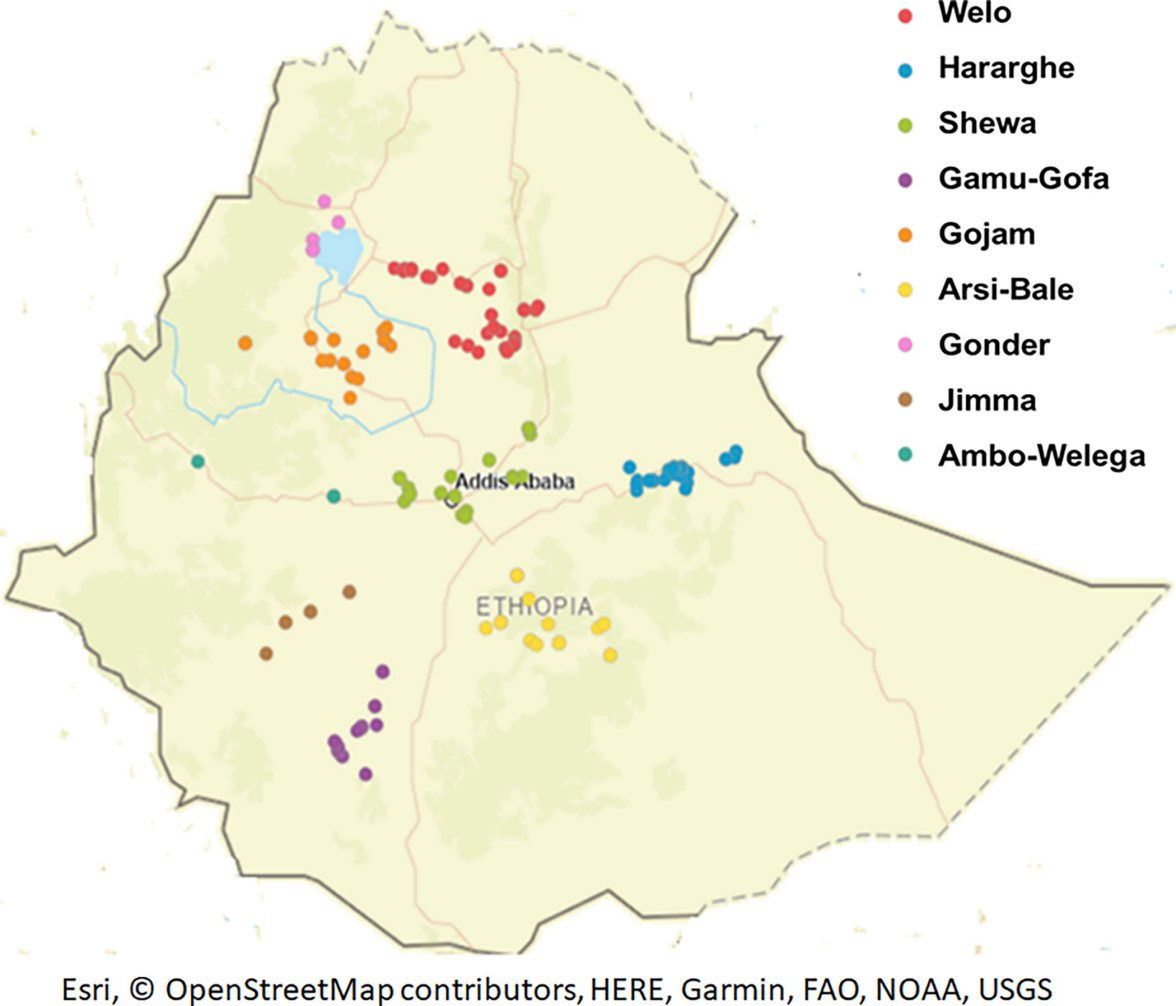
Ethiopian barley landrace accessions grouped by their geographical collection areas. Ethiopian boundary and geo-positions are indicated. Filled circles represent the 239 Ethiopian landraces collected at sometimes overlapping positions. Geographical positions are also detailed in S1 Table. The map was constructed using the online ArcGIS software suite vs. 10.8.1 provided by Esri.

A panel of 260 Ethiopian barley accessions was analyzed in this study (S1 Table). The 239 landrace accessions were obtained from the Ethiopian Biodiversity Institute (EBI). These were collected from diverse agro-ecological zones and represent different geographical regions of Ethiopia. The geographical locations in which the landraces were collected are shown in Fig 1, which is constructed using the GPS data of the collection area using a free ArcGIS online web program (https://www.arcgis.com) provided by Esri [51]. Additionally, 21 barley breeding lines were obtained from the national barley improvement program of the Holetta Agricultural Research Center (HARC).

### Genotyping

Three seeds from each of the 260 accessions were grown in the greenhouse at day (16h)/ night (8h) temperatures of 20–22°C/17-19°C as described by [52] in multipot trays filled with Einheitserde ED73 soil containing 14% N, 16% P_2_O_5_ and 18% K_2_O in kg/m3 (H. Nitsch & Sohn GmbH & Co. KG, Germany). When plants had grown to the two to three leaf stage, leaf samples with an approximate size of 300 mg were taken from a single plant for genotyping. The genomic DNA was extracted using a modified CTAB (cetyltrimethylammonium bromide) method [53] and genotyped using the barley Illumina 50k iSelect SNP array [38] at TraitGenetics GmbH, Gatersleben, Germany.

An initial set of 40,387 markers was successfully extracted from genotyping. 10,644 SNP markers were obtained, after removing all monomorphic markers and imputation using Beagle [54] followed by final filtering using thresholds of 5% missing values, 3% minor allele frequency, and 12.5% heterozygous SNPs. A total of 983 highly informative markers were kept, using the software PLINK 1.9 (http://www.cog-genomics.org/plink/1.9/) [55], which uses the markers physical distances as well as pair wise linkage disequilibrium (LD) between adjacent markers to prune-in SNPs in strong LD, with unbiased representation along the genome.

### Population structure

The 983 highly informative SNP markers were used for population structure and genetic diversity analysis. The population structure was calculated using the Structure software v.2.3.4 [56]. Computation of Bayesian statistical models was conducted by the Markov Chain Monte Carlo (MCMC) method based on 50,000 iterations following discard of 50,000 “burn-in” iterations. The web-based Structure Harvester software v0.6.94 (http://taylor0.biology.ucla.edu/structureHarvester/) [57] was used to identify the best probable number of subpopulation (k-value) according to [58]. From the best k-value, out of 10 replications the replication with the highest likelihood (mean LnP(K)) value was used as an inferred population cluster. The estimated membership coefficient of each accession was used to assign it to different clusters estimated by STRUCTURE based on the highest inferred cluster values. Principal coordinate analysis (PCoA) was applied to plot the population structure using the DARwin 5.0 software [59] based on the SNP matrix data.

### Genetic diversity

The 983 highly informative SNP markers were used for genetic diversity analysis. AMOVA was performed based on the number of genetically distinct clusters obtained from the structure analysis. Information about genetic variation within and between clusters based on PhiPT (analogue of fixation index (F_ST_)) were obtained from the analysis using the GenAlEX 6.5 software plugin for Excel [60]. The neighbor-joining tree, which is constructed based on the genetic distance of accessions [48], was created using the DARwin 5.0 software [59] to graphically demonstrate the presence of genetic distance between the subpopulations.

The genetic variance within and between clusters was calculated using the following formulas:

$$PhiPT=\frac{AP}{\left(WP+AP\right)}$$

Where PhiPT is the genetic differentiation within and between clusters; AP is the estimated variance among clusters, and WP is the estimated variance within clusters.

Detection of first-generation of migrants was conducted by converting the structure file to ’Fstat’ file using “PGDSpider 2.1.1.5” program [61], and analyzed by GeneClass2.0.h computer program [62] using Monte Carlo resampling methods [46].

Genetic diversity indices i.e. Shannon’s information index (I), expected heterozygosity (He), unbiased expected heterozygosity (uHe), and percentage of polymorphic loci (PPL) were also calculated using frequency based analysis in the GenAlEX software [60]. Additionally, the Mantel test, which is used to estimate the gene flow by correlating the genetic distance with the spatial distance, i.e. GPS data in our case, was performed to get information on the genetic divergence across the geographical distance using the GenAlEX software [60].

## Results

### SNP analyses

**Fig 2:**
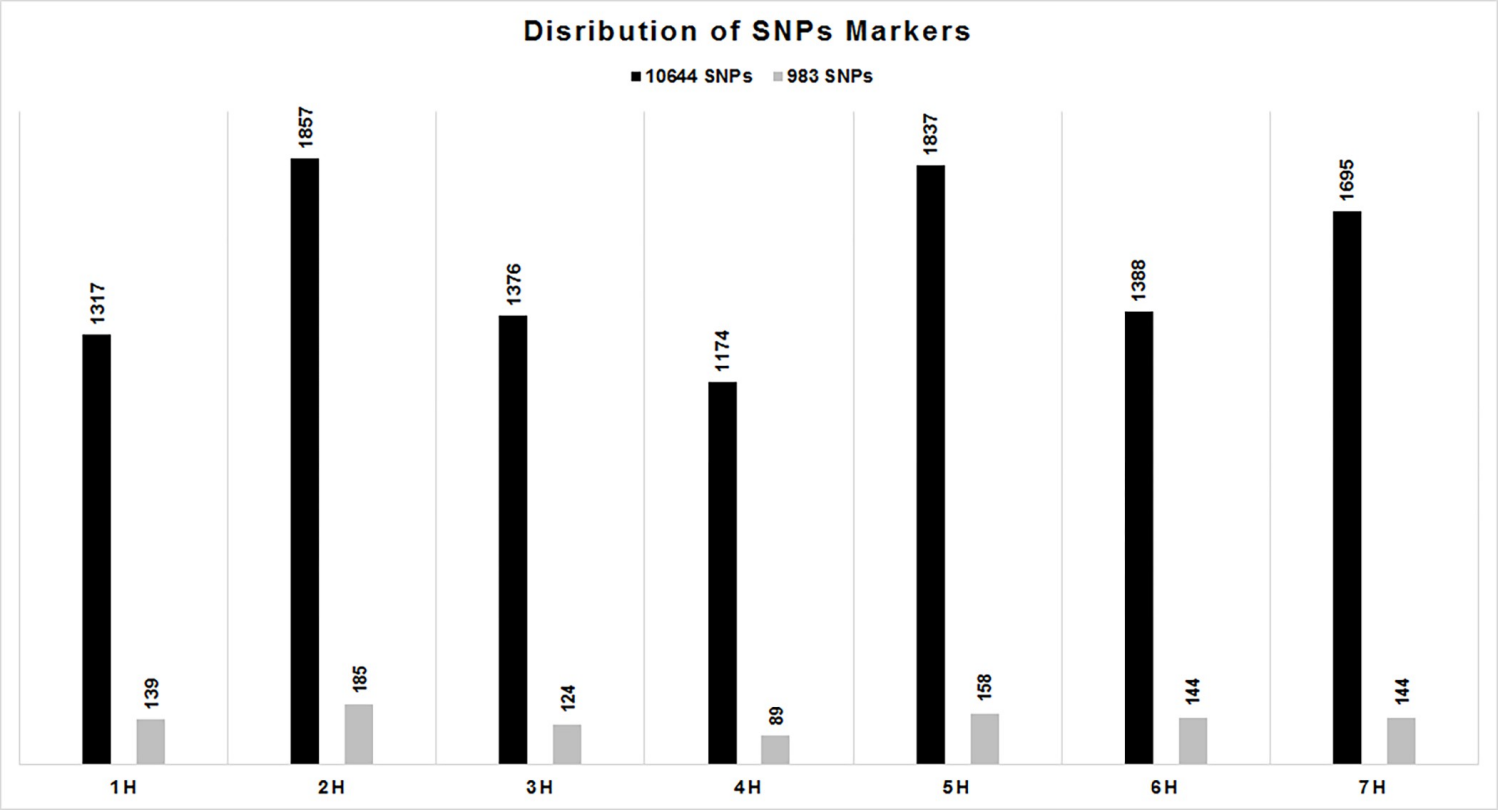
Distribution of filtered (10,644) and highly informative (983) SNPs across the barley chromosomes.

From 43,461 scorable SNPs markers of the 50k iSelect SNP array [36]; 40,387 (92.9%) SNPs markers were successfully extracted in this experiment. However, 19,028 (47.1%) markers were immediately removed as monomorphic markers. From the remaining 21,355 markers, 10,767 SNPs markers (26.7% of the extracted set of markers) were removed by filtering for 3% minor allele frequency. Out of the 10,644 SNP markers, which were obtained after filtering, the highest number of markers was located on chromosome 2H (1857), and the least markers on chromosome 4H (1174). Similarly, for the 983 highly informative markers the highest number of markers was obtained for chromosome 2H (185), and the least for chromosome 4H (89) (Fig 2). The distribution of the markers revealed that most markers in the centromeric region were pruned-out, and the majority of the highly informative markers is located in the telomeric regions of all seven chromosomes (S1 Fig).

### Population structure analysis

**Fig 3:**
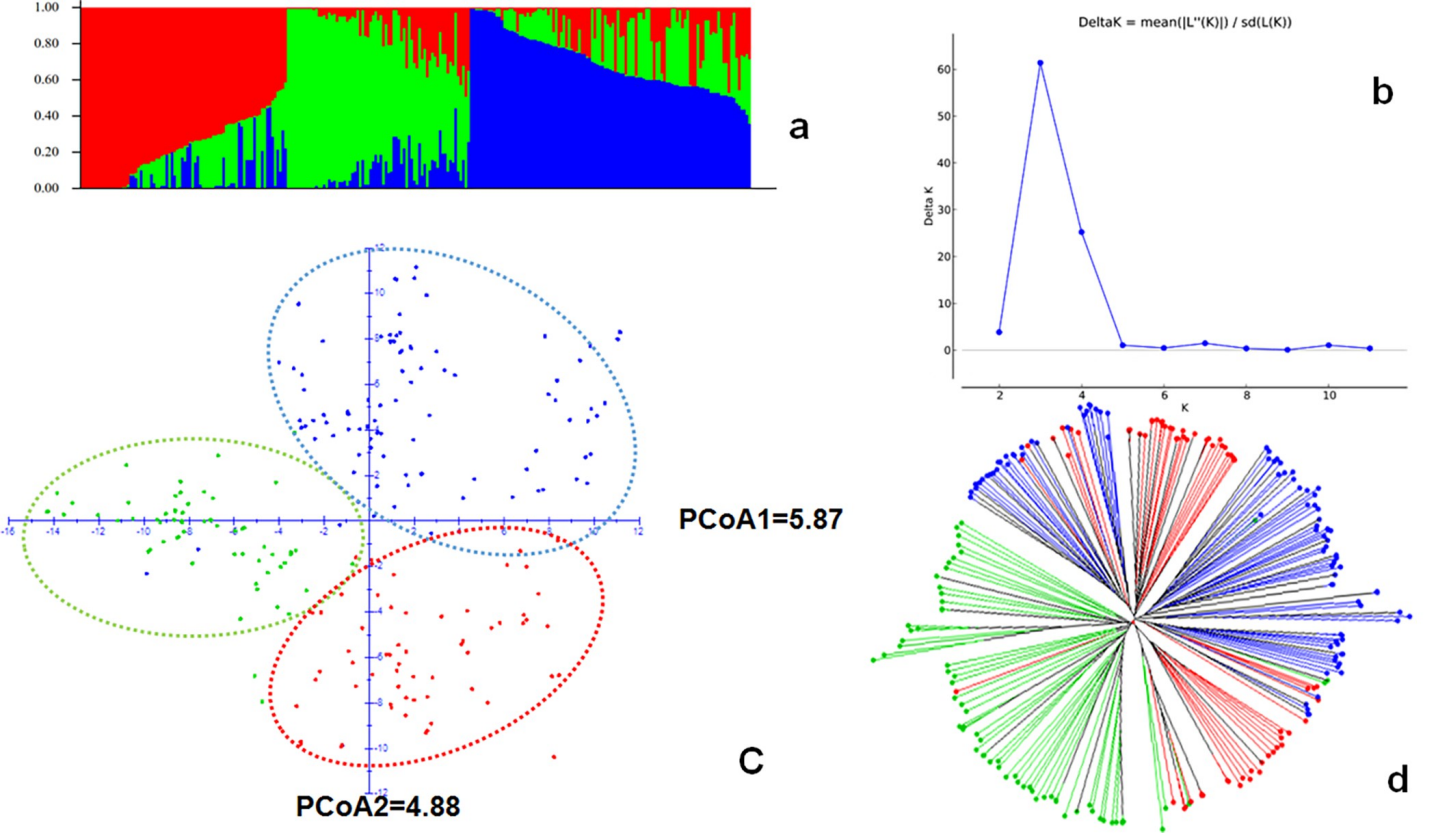
Population structure analysis for the 260 Ethiopian barley accessions. a) bar plot for estimated population structure of 260 Ethiopian barley accessions based on inferred three clusters (red = cluster 1, green = cluster 2, and blue = cluster 3); b) Structure harvester Delta K (ΔK) = 3; c) results of principal coordinate (PCoA) analysis, accessions were assigned based on their highest probability of inferred clusters; and d) weighted neighbor-joining tree for the structured subpopulations (red = cluster 1, green = cluster 2, and blue = cluster 3).

Analysis of the population structure based on 983 SNP markers identified the best probable number of the subpopulation based on k-value at K = 3, which therefore has been selected as an optimal number of inferred genetically defined clusters (Fig 3A and 3B). According to the three genetically distinct clusters, cluster 1 consists of 80 accessions (30.8%), cluster 2 consists of 71 accessions (27.3%) and cluster 3 consists of 109 accessions (41.9%) out of the total of 260 accessions (Table 1). The average membership coefficient of the geographically defined populations indicated that Welo and Shewa population can be explained by cluster 1 and 2, respectively; whereas Gonder, Gamo-Gofa, and Jimma population were explained by cluster 3 (Table 2). When each member of a geographically defined population was re-assigned based on their highest probability value of the inferred clusters, 56% and 66% of Welo and Shewa accessions were clustered in genetically distinct cluster 1 and 2, respectively. Similarly, 88%, 86%, and 71% of Gonder, Gamo-Gofa, and Jimma accessions were grouped in the genetically distinct cluster 3, respectively (Table 1). Furthermore, 75% of the Ambo-Welega population was also assigned to cluster 3, but the low number of accessions has to be taken into account. Principal coordinate analysis (PCoA) indicated that PCoA1 and PCoA2 explained 5.87% and 4.88% of the variation, respectively. Despite these values being rather low, the high genetic variation within the set of accessions is reflected by the inferred three clusters (Fig 3C).

**Table 1:** Distribution of the Ethiopian barley accessions grouped by their geographical origin and based on the three genetically distinct clusters.

Geographically defined subpopulations	Total accessions	Percentage of accessions in genetically distinct clusters					
		Cluster 1		Cluster 2-		Cluster 3	
		Number	%	Number	%	Number	%
Gonder	8	1	12.5	0	0.0	7	87.5
Arsi-Bale	19	4	21.1	4	21.1	11	57.9
Shewa	38	6	15.8	25	65.8	7	18.4
Ambo-Welega	4	0	0.0	1	25.0	3	75.0
Gojam	28	6	21.4	14	50.0	8	28.6
Welo	59	33	55.9	13	22.0	13	22.0
Gamo-Gofa	28	2	7.1	2	7.1	24	85.7
Jimma	7	1	14.3	1	14.3	5	71.4
Hararghe	48	20	41.7	5	10.4	23	47.9
HARC	21	7	33.3	6	28.6	8	38.1
**Total**	**260**	**80**	**30.8**	**71**	**27.3**	**109**	**41.9**

**Table 2:** Average membership coefficient of Ethiopian geographically defined subpopulations based on the three genetically distinct clusters.

**Geographically defined subpopulations**	**Total accessions**	**Average membership coefficient of the subpopulations in the three genetically distinct clusters**		
		**K1**	**K2**	**K3**
Gonder	8	0.182	0.210	0.609
Arsi-Bale	19	0.202	0.355	0.444
Shewa	38	0.254	0.564	0.181
Ambo-Welega	4	0.221	0.421	0.359
Gojam	28	0.208	0.475	0.316
Welo	59	0.516	0.272	0.212
Gamo-Gofa	28	0.133	0.261	0.606
Jimma	7	0.145	0.256	0.600
Hararghe	48	0.437	0.161	0.402
HARC	21	0.334	0.376	0.290
**Total**	**260**	**0.326**	**0.329**	**0.344**

### Analysis of molecular variance (AMOVA)

AMOVA analysis was conducted based on the three genetically distinct clusters obtained through the analysis of population structure. The results revealed that variation within a cluster was accounting for higher variation (89%) than the variation among clusters (11%). The genetic differentiation was moderate (PhiPT = 0.11) with statistical significance at p < 0.001 (Table 3). Based on Monte Carlo resampling model, five accessions with p<0.01 were detected as first-generation migrants. Three were from genetically distinct cluster 1, of which two and one of the accessions are likely to be immigrant from genetically distinct cluster 2 and 3 respectively. The remaining two were from genetically distinct cluster 2, of which each of the accessions are likely to be immigrant from genetically distinct cluster 1 and 3 (S2 Table).

**Table 3:** Analysis of molecular variance (AMOVA) for the Ethiopian barley accessions for the three genetically distinct clusters; PhiPT values for the total population.

Source	Degree of Freedom	Sum of square	Mean square	Estimated variance	Percentage of variation	PhiPT
**Among Populations**	2	6,576.5	3,288.2	35.3	11%	0.11**
**Within Populations**	257	73,143.0	284.6	284.6	89%	
**Total**	**259**	**79719.5**		**319.9**	**100%**	

** p-value < 0.001.

### Genetic diversity

The study of the genetic diversity indices of the three genetically distinct clusters indicate, that cluster 3 is more diverse than the other two clusters with values of I = 0.47, He = 0.31, uHe = 0.31, PPL = 99.1%, followed by cluster 2 (I = 0.43, He = 0.28, uHe = 0.28, PPL = 95.9%) while cluster 1 is the least divers one (I = 0.39, He = 0.26, uHe = 0.26, PPL = 88.2%) (Table 4).

**Table 4:** Genetic diversity indices for the genetically distinct clusters.

Genetically distinct clusters	N	I	He	uHe	PPL
**1**	80	0.39	0.26	0.26	88.2%
**2**	71	0.43	0.28	0.28	95.9%
**3**	109	0.47	0.31	0.31	99.1%

“N” for number of observations, “I” for Shannon’s information index, “He” for expected heterozygosity, “uHe” for unbiased heterozygosity, and “PPL” for percentage of polymorphic loci.

Based on the results of pairwise PhiPT value, there is a moderate genetic differentiation between the subpopulations. The results indicate that the variation between genetically distinct cluster 1 and 2 is relatively larger (0.13) than between the other populations (S3 Table).

**Fig 4:**
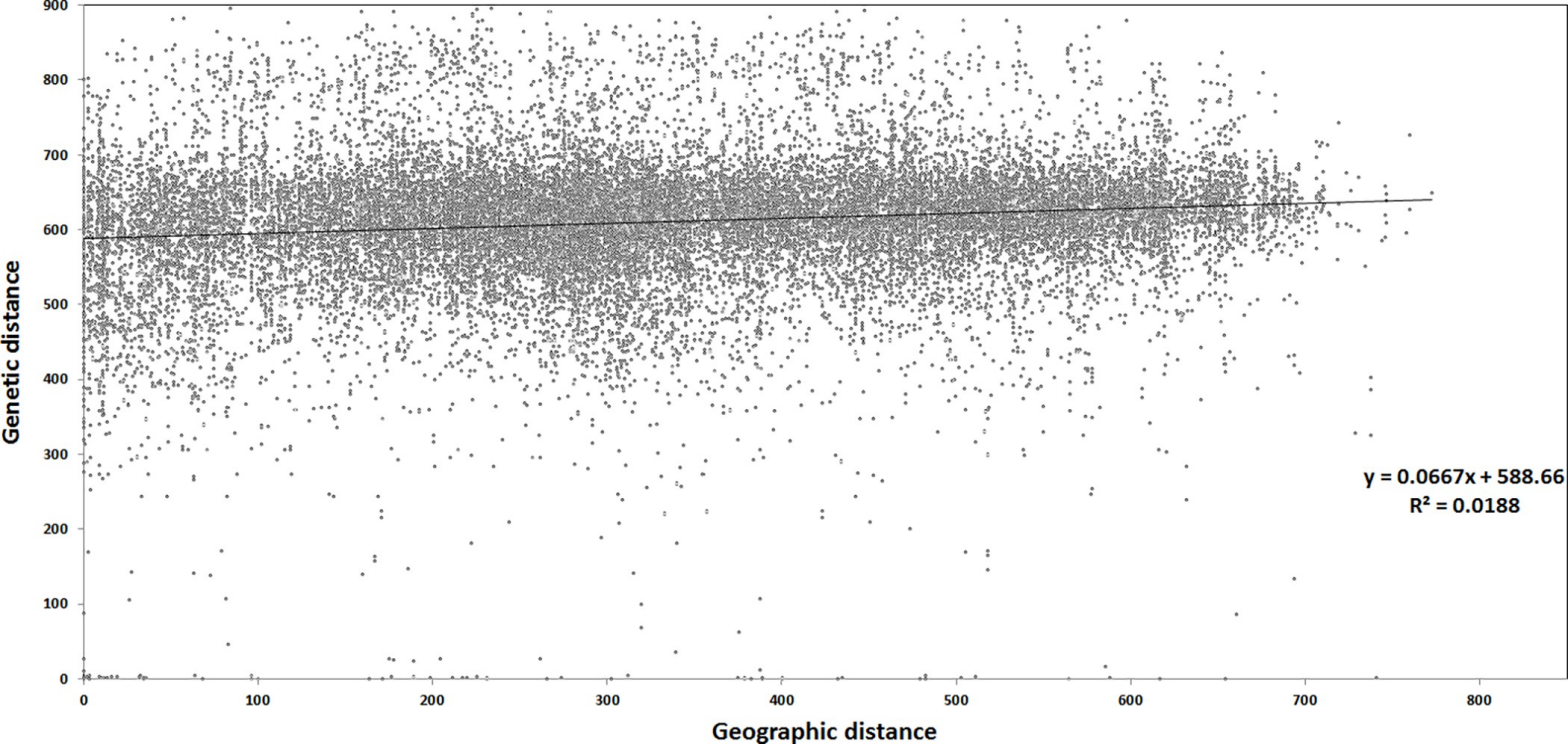
Mantel test for the 239 landraces based on the relationship between the genetic distance and the geographic distance based on GPS data.

The Mantel test, which is used to demonstrate the presence of spatial population structure indicated that the accessions were poorly structured, based on the GPS data of sampling with an R-squared value of 0.019 (Fig 4).

## Discussion

From 43,461 scorable SNPs markers of the 50k iSelect SNP array [38]; the final number of SNPs markers (10,644) in our study was quite small compared to previous reports of 39,733 SNPs [63]; 33,818 SNPs [64] and 37,242 SNPs [65]. This may be explained by the under-representation of Ethiopian genotypes during the development of the 50k SNP array [38].

The distribution across the barley genome of the SNPs markers obtained after filtering was compared with the one of the 50k SNP array [38]. The genome regions containing the first and the second highest number of SNP markers were on chromosome 5H (8123) and chromosome 2H (7227) for the 50k SNP array development, whereas in our study the first and second highest representation were recorded on chromosome 2H (1857) and chromosome 5H (1837). The two genome regions with the least number of SNP markers were chromosome 1H (4828) and chromosome 6H (5441) for the 50k SNP array, while in this study chromosome 4H (1174) and chromosome 1H (1317) were least represented. Therefore, we considered the distribution of SNP markers along the seven barley chromosomes as similar with the 50k SNP array. A total of 983 highly informative markers, located in the telomeric regions of all seven chromosomes (S1 Fig), were kept for the population structure analyses.

According to the average membership coefficient, the predefined Welo and Shewa subpopulations were classified as genetically distinct in cluster 1 and 2, respectively (Table 1). By the ratio of accessions assigned in each cluster, accessions from Gonder, Gamo-Gofa and Jimma, predefined as subpopulations, appeared to be represented by cluster 3 (Table 2). Similarly, the average membership coefficient of the Gonder, Gamo-Gofa, and Jimma (Table 1) populations clearly suggested that they are members of cluster 3. [66] reported that landraces obtained from Shewa, Gonder, and Gojam have had minimum admixture, whereas landraces obtained from Arsi-Bale, Harerghe, and Welo were showing the highest ratio of admixture. Accordingly, in our study, landraces from Gonder, and Shewa were grouped in cluster 1 and 3 respectively; and Arsi-Bale and Harerghe were not defined by any cluster (Table 2).

Estimation of the population structure along the geographical and agro-ecological arrangement gives an important view on the pattern of population structure. In Ethiopia, studies conducted on different cereal crops highlighted the presence of higher genetic variation within geographical locations and altitude ranges for barley [50, 67, 68], durum wheat [69–71], and sorghum [72]. Similarly, the presence of minimum geographical structure was observed using the Mantel test in this study (Fig 4). This may be due to the fact that accessions from distantly located regions, i.e. Gonder, Jimma and Gamo-Gofa are grouped in cluster 3. Further analysis of AMOVA based on the agro-ecological zones of the accessions as a predefined subpopulation provided only 3% variation between agro-ecologies (S4 Table), although the variation between genetically distinct clusters was 11% (Table 3). On the contrary, previous genetic diversity studies on Ethiopian barley landraces suggested that the landraces’ population structure is dependent on the altitudinal gradient; which is mainly used for the classification of Ethiopian agro-ecologies [14, 21, 73, 74], but of which a minimum of variation explained was found in the current study (S4 Table).

The influence of altitudinal gradient on population structure was reported as important by [14, 21, 73, 74], in the contrary other studies [50, 66–68] reported its influence as minimum for the formation of population structure. Although our study demonstrated the impact of altitudinal gradient as minimum, we carefully examined the previous studies, and the accessions’ passport data; to strengthen our findings.

Previous studies [14, 21, 73, 74], that reported the influence of altitudinal gradient on population structure, were conducted using either one or two provinces of Ethiopia; whereas studies conducted based on landraces collected from several provinces [50, 66–68], revealed the minimum impact of altitude gradient in the formation of genetic cluster. Therefore, the presence of less province representation in the samples might be influenced the outcome of the results.

To proof the impact of altitude gradient in specific province; Shewa and Welo subpopulations were selected, as they can be defined by cluster 2 and 1 respectively (Table 1). The landraces of these locations were classified based on their sources of agro-ecological zones, and in both locations 25 (66%) and 32 (52%) of their total landraces are from ‘Cool moist mid highlands (M4)’ agro-ecology zone respectively (S1 Table). These M4 landraces were further assigned in to the three genetically distinct clusters; and 16 (64%) and 17 (53%) of the M4 landraces of Shewa and Welo were assigned into cluster 2 and 1 respectively, which should be greater than the total amount of their populations assigned to cluster 2 and 1 respectively (Table 1), to confirm the importance of agro-ecological zone for the formation of structured population.

As mentioned by [20, 21], the landraces at hand of Ethiopian farmers are genetically variable. Similarly, when seed increased of the EBI accessions planting materials were conducted, morphological different plants was observed in 19 EBI accession code, which finally resulted in 39 different accessions of the study material (S1 Table). The presence of population genetic structure in their descendant accession was evaluated, the result revealed the descendants of 6 of 19 EBI accessions codes (32%) are assigned into different genetic cluster (S1 Table), which indicate the presence of uneroded genetic structure even at the farmer level. If such level of genetic structures is available at the farmer hand, the presence of higher level of genetic structures is expected within the altitudinal gradient.

Therefore, as mentioned by [66] the climate condition have weak association with structured populations of Ethiopian barley landraces, but in the contrary to [66] study the geographic locations slightly contributed to the variation among the structured populations.

The overall population genetic differentiation (PhiPT) value is (0.11) indicating the presence of moderate differentiation between the genetically clustered subpopulations [43]. Similarly, the pairwise PhiPT value between clusters ranges from 0.10 between cluster 1 and 3 to 0.13 between cluster 1 and 2 (S3 Table). The presence of moderate genetic differentiations between the different genetically distinct clusters hints to the exchange of adaptive traits among them [28, 75]. A total of five accessions were detected as first-generation migrants in the study, i.e. 1.92%. Although genetically distinct cluster 3 didn’t have any immigrants in its population, there are two accessions originally migrated from this cluster to cluster 1 and 2 (S2 Table), which indicate the direction of migration from this cluster to others is more frequent than other [76]. Similarly, the pairwise PhiPT indicated that cluster 3 have the least value to differentiated genetically from other genetically distinct (S3 Table), as it possessed extra shared markers in migrate population [76].

The presence of weak geographical or agro-ecological structure for Ethiopian barley landraces [50, 67, 68] may be explained by the exchange of important adaptive genetic traits between different genetically distinct clusters. The 21 breeding lines used in the study are proportionally distributed in the three clusters (Tables 1 and 2), which is also an indicator, that the national breeding program is introducing important adaptive traits from landraces in new varieties. The Mantel test (Fig 4) also revealed the presence of higher gene flow between the farthest locations, which may contribute to a wider adaptation of Ethiopian landraces.

From the three genetically distinct clusters, cluster 1 is explained by the Welo predefined subpopulation. [77] described that around the Welo location barley is an important crop, and farmers conserve the landraces for different reasons, such as for their suitability to use it for short and long rainy seasons (maturity), yield potential, tolerance to water logging, frost and low soil fertility, social preference (taste and visual appearance), and storability. Furthermore, barley is also used as a main dish (to prepare *injera*, and bread) in this area, and a special dish and beverage (*tihlo* and *korefe*), which are exclusively prepared from barley, are commonly consumed in this area [77]. Thus, another assumption for the formation of this genetically clustered population may be related to the landraces quality to prepare staple food as well as special dishes and beverages.

Cluster 3 mainly contains landraces from Gamo-Gofa, and the production of barley in Gamo-Gofa is mainly on highlands with an altitude higher than 2,500 meter above sea level [78]. Such highland topographies are characterized by having low road access to connect with nearest commercial cities. As a result, the diversity in such areas will be kept unchanged. Accordingly, studies suggested an increased market access in the community contributing to an increase in crop diversity [79, 80]. In our study, the presence of low market access likely contributed to the grouping of 86% of Gamo-Gofa accessions in cluster 3. Although farmers varieties selection criteria in Gamo-Gofa are similar to other locations, barley is not served as main dish in the region and usually used to prepare special dishes and beverage (local beer) during a festive holiday and special occasions [81]. We therefore assume that the farmers selection criteria for varieties may be based on the end use of the product, and consequently landraces in cluster 3 might be related with such quality traits.

Shewa is located in the central part of Ethiopia, with best road facilities, and high consumer demand. Farmers usually produce barley for home consumption and market; and [82] reported that farmers produce barley as it is adapted very well comparing to other cereal crops to the low fertility soil in this region. Barley is used in this region to prepare local liquor and local beers, which have great demand for market. Additionally farmers produce suitable landraces to prepare the main dish (*injera*) [82]. A significant reduction in the number of farmer’s varieties comparing to the previous time was reported in Shewa [83] due to socio-economic and environment related reasons. Such genetic erosion may not just be a recent history in the region, but might also be present in the previous decades, which is ultimately narrowing the genetic bases of the landraces in this area. The result obtained from weighted neighbor-joining tree (Fig 3D) and the pairwise PhiPT (S3 Table) indicated that cluster 2 derived from slightly different predecessor families, in comparison to cluster 1 and 3 which are closer related. Therefore, the remaining landraces around Shewa with a narrow genetic base may be mostly related to cluster 2 (Fig 3D, Table 2).

Cluster 3 is a diverse cluster based on the results of genetic indices (Table 4). 86% of accessions from Gamo-Gofa are assigned to this cluster and [84] also described that landraces obtained from Gamo-Gofa region have higher diversity index compared to other regions. On the contrary, landraces from Gonder, which are also grouped in cluster 3, have been described for having least diversity in that study.

## Conclusions

Genetic structure and diversity of 260 Ethiopian barley landraces, comprising 239 accessions from EBI, and 21 barley breeding lines of the national barley improvement program of the HARC, were investigated based on data obtained from the barley 50k iSelect SNP array (S5 Table). The presence of higher rates of monomorphic markers with minor allele frequency less than three seems characteristic for Ethiopian barley accessions compared with other barley collections from the world. AMOVA revealed the existence of high genetic diversity within genetically distinct populations in comparison to the genetic diversity between genetically distinct populations. This may be due to the minimum geographical structure of landraces and the presence of higher gene flow between accessions originated from distant geographic locations. The use of barley for different food recipes and beverages may also play a role in the genetically clustered population structure as [15] described the use of different barley types for different purposes by the society of different regions. However, further analysis based on the nutritional quality of each landrace in specific geographical locations may be required. Our results will support the strategic collection and exploitation of the existing genetic resources of Ethiopian barley landraces, and will help improving farm management of subsistence farmers through the dedicated utilization of genetic resources in the near future.

## Supporting information

S1 Fig Physical map distribution of SNP markers across the seven barley Chromosomes. A: Filtered 10,644 SNP markers; B: Highly informative 983 SNP markers.
(TIF)

S1 Table Geographical location based on GPS and agro-ecological zones of the Ethiopian barley landrace. Accessions descended from similar ‘EBI accession code’ are highlighted by yellow color.
(XLSX)

S2 Table First-generation migrants from genetically distinct clusters. (DOCX)

S3 Table Pairwise correlation matrix for genetic differentiation (PhiPT). (DOCX)

S4 Table Molecular variance (AMOVA) for the Ethiopian barley accessions based on the 14 defined agro-ecological zones; genetic differentiation (PhiPT) of the total population. (DOCX)

S5 Table Raw ‘hapmap’ data file containing the 983 informative SNPs for the Ethiopian barley landraces used in the study. (XLSX)
