# Supplementary figures and images for: The genetic diversity of Ethiopian barley genotypes in relation to their geographical origin
Source: PLoS One. 2022 May 27;17(5):e0260422. doi: 10.1371/journal.pone.0260422 (PMC9140232; doi:10.1371/journal.pone.0260422)

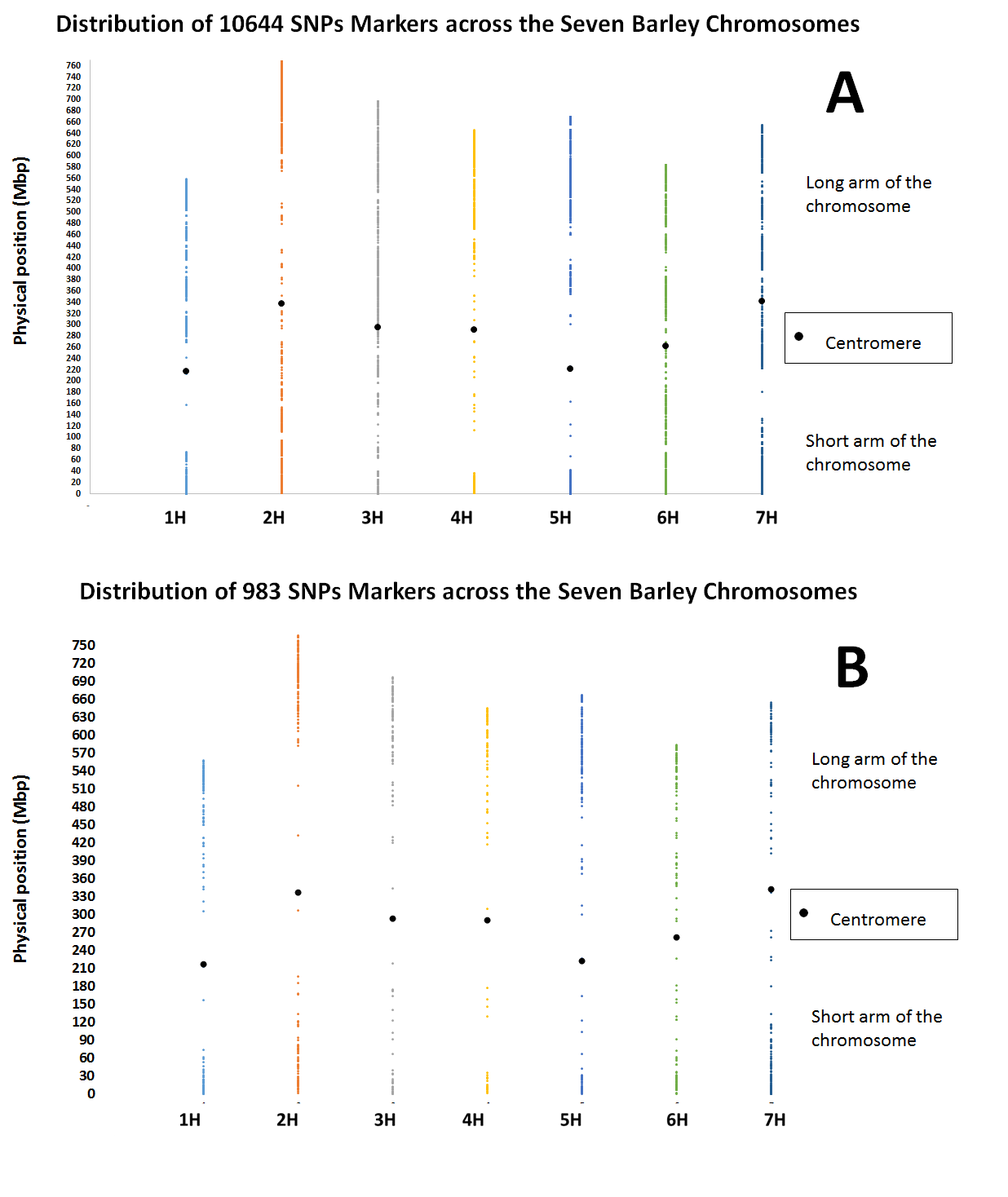

Supplement: S1 Fig — A: Filtered 10,644 SNP markers; B: Highly informative 983 SNP markers. (TIF) [file pone.0260422.s001.tif]
