# Supplementary material for: The genetic diversity of Ethiopian barley genotypes in relation to their geographical origin
Source: PLoS One. 2022 May 27;17(5):e0260422. doi: 10.1371/journal.pone.0260422 (PMC9140232; doi:10.1371/journal.pone.0260422)
Supplement: S2 Table — (DOCX) [file pone.0260422.s003.docx]

**S2 Table. First-generation migrants from** **genetically distinct clusters**

| **Genetically distinct** | | **Total migrants** | | **Total number of migrants likely originated from genetically distinct clusters** | | |
| --- | --- | --- | --- | --- | --- | --- |
| **Clusters** | **Num. of members** | **Num.** | **Perc.** | **1** | **2** | **3** |
| **1** | **80** | 3 | 3.75 | . | 2 | 1 |
| **2** | **71** | 2 | 2.82 | 1 | . | 1 |
| **3** | **109** | 0 | 0 | 0 | 0 | . |
| **Total** | **260** | **5** | **1.92** | **1** | **2** | **2** |

**Remark:** Num. = Number; Perc. = Percentage from total number of members
