# Supplementary material for: The genetic diversity of Ethiopian barley genotypes in relation to their geographical origin
Source: PLoS One. 2022 May 27;17(5):e0260422. doi: 10.1371/journal.pone.0260422 (PMC9140232; doi:10.1371/journal.pone.0260422)
Supplement: S3 Table — (DOCX) [file pone.0260422.s004.docx]

**S3 Table. Pairwise correlation matrix for genetic differentiation (PhiPT).**

| **Subpopulation** | **1** | **2** | **3** |
| --- | --- | --- | --- |
| **1** |  | 0.13 | 0.10 |
| **2** |  |  | 0.11 |
| **3** |  |  |  |
