# Supplementary material for: The genetic diversity of Ethiopian barley genotypes in relation to their geographical origin
Source: PLoS One. 2022 May 27;17(5):e0260422. doi: 10.1371/journal.pone.0260422 (PMC9140232; doi:10.1371/journal.pone.0260422)
Supplement: S4 Table — (DOCX) [file pone.0260422.s005.docx]

**S4 Table. Molecular variance (AMOVA) for the Ethiopian barley accessions based on the 14 defined agro-ecological zones; genetic differentiation (PhiPT) of the total population.**

| **Source** | **Degree of freedom** | **Sum of square** | **Mean square** | **Estimated variance** | **Percentage of variation** | **PhiPT** |
| --- | --- | --- | --- | --- | --- | --- |
| **Among Populations** | 13 | 5,607.4 | 431.3 | 7.7 | 3% | 0.03 |
| **Within Populations** | 246 | 74,112.1 | 301.3 | 301.3 | 97% |  |
| **Total** | **259** | **79,719.5** |  | **309.0** | **100%** |  |
